# RNAstructure: software for RNA secondary structure prediction and analysis

**DOI:** 10.1186/1471-2105-11-129

**Published:** 2010-03-15

**Authors:** Jessica S Reuter, David H Mathews

**Affiliations:** 1 Department of Biochemistry & Biophysics and Center for RNA Biology, University of Rochester Medical Center, 601 Elmwood Avenue, Box 712, Rochester, NY 14642, USA

## Abstract

**Background:**

To understand an RNA sequence's mechanism of action, the structure must be known. Furthermore, target RNA structure is an important consideration in the design of small interfering RNAs and antisense DNA oligonucleotides. RNA secondary structure prediction, using thermodynamics, can be used to develop hypotheses about the structure of an RNA sequence.

**Results:**

RNAstructure is a software package for RNA secondary structure prediction and analysis. It uses thermodynamics and utilizes the most recent set of nearest neighbor parameters from the Turner group. It includes methods for secondary structure prediction (using several algorithms), prediction of base pair probabilities, bimolecular structure prediction, and prediction of a structure common to two sequences. This contribution describes new extensions to the package, including a library of C++ classes for incorporation into other programs, a user-friendly graphical user interface written in JAVA, and new Unix-style text interfaces. The original graphical user interface for Microsoft Windows is still maintained.

**Conclusion:**

The extensions to RNAstructure serve to make RNA secondary structure prediction user-friendly. The package is available for download from the Mathews lab homepage at http://rna.urmc.rochester.edu/RNAstructure.html.

## Background

The prediction of RNA structure has received increasing attention over the last decade as the number of known functional RNA sequences, called non-coding RNA (ncRNA), has increased [1]. These new ncRNA sequences range in size from microRNAs to Xist [2,3]. They serve numerous roles, from modulating gene expression [4-6] to catalyzing reactions [7,8].

One of the first steps to understanding the mechanism of action of an RNA is to determine its structure [9]. Secondary structure, defined as the set of canonical base pairs (AU, GC, and GU), can be determined using comparative analysis if a large number of sequences are available [10,11]. In comparative analysis, base pairs are determined when they are conserved in multiple sequences and instances of compensating base pair changes occur. Compensating base pair changes demonstrate the conservation of structure in spite of sequence not being conserved, for example a GC base pair in one sequence being replaced by a homologous AU pair in another sequence. Comparative analysis, however, requires both significant user input and a large number of homologous sequences that can be aligned.

As an alternative to comparative analysis, the secondary structure of an RNA can be predicted for a single sequence using thermodynamics [9]. The thermodynamic methods are based on nearest neighbor rules that predict the stability of a structure as quantified by folding free energy change [12-14]. Often, structure prediction is accomplished by finding the lowest free energy structure, which is the single most probable structure in a folding ensemble [15]. Alternatively, structures can be sampled from the Boltzmann ensemble and a centroid, i.e. representative structure, determined [16,17]. Another alternative method for structure prediction is the prediction of a structure with the highest sum of pairing probabilities, called the maximum expected accuracy structure [18,19].

Single sequence secondary structure prediction is reasonably accurate. On average, for sequences of fewer than 700 nucleotides, the accuracy of predicting known base pairs is as high as 73% [14]. The accuracy, however, benchmarked lower when longer sequences were included [20,21]. Additional sources of information can be used to improve accuracy. For example, base pair probabilities can be determined using a partition function and highly probable pairs are more likely to be correctly predicted pairs [22]. Alternatively, using two or more homologous sequences to determine a conserved structure can result in significantly more accurate structure prediction [23-27]. Experimental data, such as enzymatic cleavage [13], chemical mapping [14], oligonucleotide array binding [28], SHAPE [29], and NMR data [30] can all be used to improve structure prediction accuracy.

In addition to structure prediction, the thermodynamic methods can be applied to other problems. For example, antisense oligonucleotide and siRNA design can be improved using thermodynamic predictions of self-structure in the oligonucleotides and target [31-36]. Sequences can be designed to fold to a specific structure [37,38]. Reverse-PCR primers can be designed to avoid self structure in the template that could prevent hybridization [39]. Novel types of ncRNAs can be found in genomes on the basis of folding stability [40-42].

In this contribution, the RNAstructure software package is described. RNAstructure first appeared in the literature in 1998 as a secondary structure prediction package [43]. At that time, it contained a method to predict the lowest free energy structure and a set of low free energy structures [44,45]. It was subsequently expanded to include bimolecular folding and hybridization thermodynamics with OligoWalk [13,31,33]. It was then expanded to include an algorithm for finding lowest free energy structures common to two sequences, Dynalign [23,41,46]; a partition function algorithm [22]; an alternative prediction method that can determine all low free energy structures for a sequence [28,47]; and stochastic sampling of structures [48]. It provides methods for constraining structures with enzymatic data [13], chemical mapping data [14], SHAPE [29], and NMR data [30]. Finally, recent extensions include PARTS [24], which calculates partition functions for secondary structures common to two sequences and can perform stochastic sampling of common structures [48]; MaxExpect, which finds maximum expected accuracy structures [18]; and a method for removal of pseudoknots, leaving behind the lowest free energy pseudoknot-free structure [49]. Several tutorials exist for using RNAstructure [50-52].

RNAstructure has been publicly available with a user-friendly interface for Microsoft Windows. Testing and development occur in-house using Unix/Linux text-based interfaces, but these have generally been available only upon request. The package is coded in C++. Extensive benchmarks of individual components have been published [14,18,22-24,29-31,46,48,53,54].

Here, three major extensions of RNAstructure are reported. First, a new JAVA-based graphical interface (GUI) is available. This interface functions cross-platform and binaries are available for Apple OS-X and Linux. Second, text interfaces are now available for each component of the package. These interfaces use a standard Unix syntax and include online help. Binaries for the text interfaces are available for Microsoft Windows and source code, including Makefiles, are available for download. Finally, a new class library is available for programmers who want to incorporate the RNAstructure functions into C++ programs. Each of these components is available for download and covered by the GNU Public License, version 2.

## Implementation

### C++ class library

**Figure 1:**
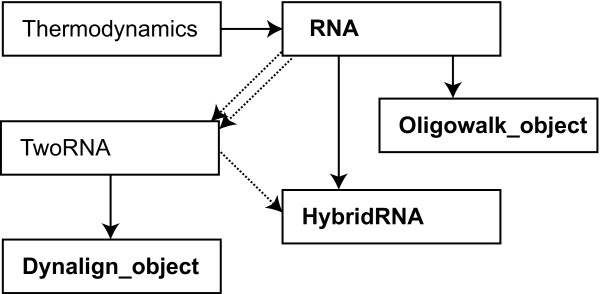
**Class inheritance diagram**. The four main classes that provide functionality, named in boldface, are RNA, Oligowalk_object, HybridRNA, and Dynalign_object. Solid lines show inheritance and the dotted lines indicate that a class contains an instance of another. TwoRNA contains two instances of RNA and HybridRNA contains an instance of TwoRNA.

The C++ class library encapsulates the I/O functions of RNAstructure and also the secondary structure prediction and analysis methods. Four main classes are provided for accessing these functions: RNA, for single sequence structure predictions; Dynalign_object, for Dynalign calculations [23]; HybridRNA, for bimolecular structure prediction [33]; and Oligowalk_object, for OligoWalk [31,33] and OligoScreen [55] calculations. Two other notable classes are utilized by inheritance by the four main classes. Thermodynamics is a class that handles reading and storage of the nearest neighbor parameters. TwoRNA is a class that contains two RNA classes and is inherited by Dynalign_object and instantiated by HybridRNA. A class inheritance diagram is provided as Figure 1.

The classes are designed to be easily included in C++ projects. The classes are compiled to Linux/Unix shared libraries, Windows dynamic link libraries, or Macintosh dynamic shared libraries. An included Makefile provides facility for this. Furthermore, the header (*.h) files are commented using Doxygen-formatted comments that facilitate the creation of a manual http://www.stack.nl/~dimitri/doxygen/. Programmers can either compile their own manuals into html or latex using Doxygen or they can refer to precompiled html manuals included in the source code and posted on the RNAstructure website.

### Text interfaces

The new text interfaces are designed to provide the features of RNAstructure for use on the command line and in scripts. Most programs available in the graphical user interfaces are provided with text interfaces. Each of these programs provides a brief description of the parameters when invoked without parameters. Additionally, invoking most programs with "-h," "-H," or "--help" will return a more detailed description of the parameters.

The text interfaces are built with ANSI-standard C++. Makefiles are included for compiling the programs in a Unix/Linux/OS X environment. Binaries for Microsoft Windows are also available for download.

The RNAstructure class library is used to implement most of the functions in the text interfaces. Therefore, the text interfaces can be used as tutorials by programmers implementing the RNAstructure algorithms in their own programs.

### JAVA Graphical User Interface

The new JAVA GUI is a cross-platform re-implementation of the RNAstructure Windows GUI. This is designed to be user-friendly to make these algorithms accessible to a large audience. Executables are available for Apple OS-X and both 32-bit and 64-bit Linux. A Makefile is provided to facilitate local compilation on Unix/Linux environments.

The JAVA GUI utilizes the new RNAstructure C++ class library to implement the algorithms. The connection between the JAVA front end and the C++ back end is made using SWIG http://www.swig.org/. SWIG wraps the C++ classes, making them accessible to JAVA. The SWIG-wrapped code is made available as part of the RNAstructure code download, so that the JAVA interface can be built on local machines that do not have SWIG installed.

### Availability of algorithms

Table 1 shows the availability of the different single-sequence structure prediction and analysis algorithms in RNAstructure. It shows the names of the text interface programs, the corresponding function name and class library, and the menu name in the JAVA and Windows GUIs. Similarly, Table 2 shows the corresponding information for the multi-sequence methods (either for common secondary structure prediction or prediction of nucleic acid hybridization).

**Table 1:** Single Sequence Methods.

Feature:	Text Interface Program:	JAVA/Windows GUI Menu Item:	Class Library and Function Name:
Free energy minimization structure prediction [14]	Fold	Fold RNA Single Strand	RNA::FoldSingleStrand

Maximum expected accuracy structure prediction [18]	MaxExpect	Predict Maximum Expected Accuracy Structure	RNA:MaximizeExpectedAccuracy

Partition function [22]	partition	Partition Function RNA	RNA::PartitionFunction

Efn2 (energy calculator) [13]	efn2	Efn2 RNA	RNA:: CalculateFreeEnergy

Free energy minimization and generation of all suboptimal structures [28,47]	AllSub	Generate All Suboptimal RNA Structures	RNA::GenerateAllSuboptimalStructures

Stochastic sampling of structures [17]	stochastic	Stochastic RNA Sampling	RNA::Stochastic

Remove Pseudoknots [49]	RemovePseudoknots	Break Pseudoknots	RNA::BreakPeudoknots

Prediction of structures with pairs above specified pairing probability threshold [22]	ProbablePair	Output Probable Structure	RNA::PredictProbablePairs

Drawing secondary structure diagrams	draw	Draw	RNA::DetermineDrawingCoordinates

NAPSS [30]	NAPSS	-	-

**Table 2:** Multiple Sequence Methods.

Feature:	Text Interface Program:	JAVA/Windows GUI Menu Item:	Class Library and Function Name:
Dynalign [23,41,46]	dynalign	Dynalign RNA	Dynalign_object::Dynalign

OligoWalk [31-33]	OligoWalk	OligoWalk	Oligowalk_object::Oligowalk

OligoScreen [55]	oligoscreen	OligoScreen	Oligowalk_object::OligoScreen

Bimolecular structure prediction with intramolecular pairs [33]	bifold	Fold RNA Bimolecular	HybridRNA::FoldBimolecular

PARTS [24,48]	PARTS	-	-

Bimolecular partition function (no intramolecular pairs) [31]	bipartition	Partition Function RNA Bimolecular	HybridRNA::PartitionFunctionBimolecular

Bimolecular structure prediction without intramolecular pairs	DuplexFold	-	HybridRNA::FoldDuplex

### Thermodynamic parameters

The algorithms implemented in RNAstructure use nearest neighbor parameters to predict the stability of secondary structures. These include both free energy change parameters at 37°C and enthalpy change parameters to allow prediction of conformation stability at an arbitrary temperature. For RNA, these parameters are those most recent parameters from the Turner group [12,14,54]. For DNA, the parameters are derived from the experimental literature [56-92]. Most algorithms for RNA structure prediction can be invoked for DNA structure prediction using the class, text interfaces, or GUIs. DNA-RNA hybridization parameters are also used by OligoWalk [93].

### Unit testing

The RNAstructure package now includes a facility for unit testing. A Makefile automates testing of the text interfaces and comparison with calculation standards. The same tests can be used to test the GUIs, but this requires manual selection of the input. Unit testing is helpful when changes are made to the algorithms to ensure that structure prediction is unchanged. Unit testing is also important when RNAstructure is installed in environments that have not been previously tested, to ensure that the installation worked correctly.

## Results

To demonstrate the utility of RNAstructure, an example of secondary structure prediction is provided, showing the input and output. The method utilized is free energy minimization and the example sequence is the 5S rRNA from *Pneumocystis carinii *[94].

**Figure 2:**
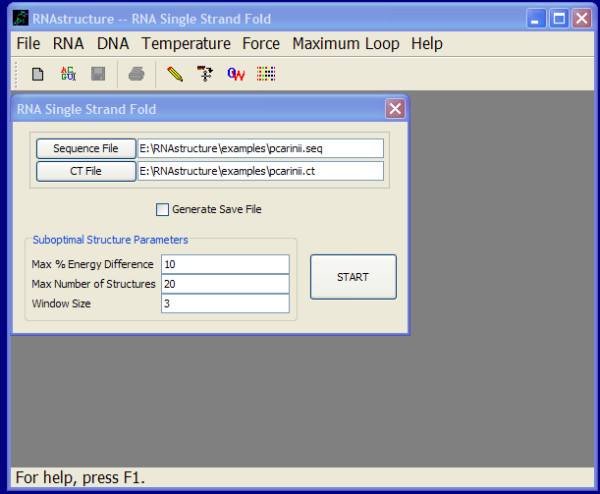
**A screenshot of the JAVA GUI for predicting the lowest free energy structure for sequence "pcarinii.seq"**.

Figure 2 shows a screenshot of the "Fold RNA Single Strand" input form from the JAVA GUI. This program predicts the lowest free energy structure for an input sequence and a set of low free energy structures called suboptimal structures. The name of the input sequence is already selected in this screenshot by having clicked the "Sequence File" button. The default output (CT) file and the default parameters for determining suboptimal structures were determined by the program. Save files can be written to accelerate the prediction of an alternative set of suboptimal structures with a subsequent calculation. This view of the GUI shows the checkbox unchecked and so no save file will be written. The three remaining parameters control the prediction of suboptimal structures. "Max % Energy Difference" and "Max Number of Structures" place limits on the number of suboptimal structures predicted, with a maximum of the percentage difference in free energy change above the lowest free energy structure or an absolute limit on the number of structures, respectively. Suboptimal structures will be generated until the percent energy difference above the lowest free energy structure is reached, 10% in this case, or 20 structures have been generated. "Window Size" determines how different the suboptimal structures must be from each other in the set [44]. Zero places no restriction and larger integers become increasingly more stringent in the number of different pairs required between structures.

**Figure 3:**
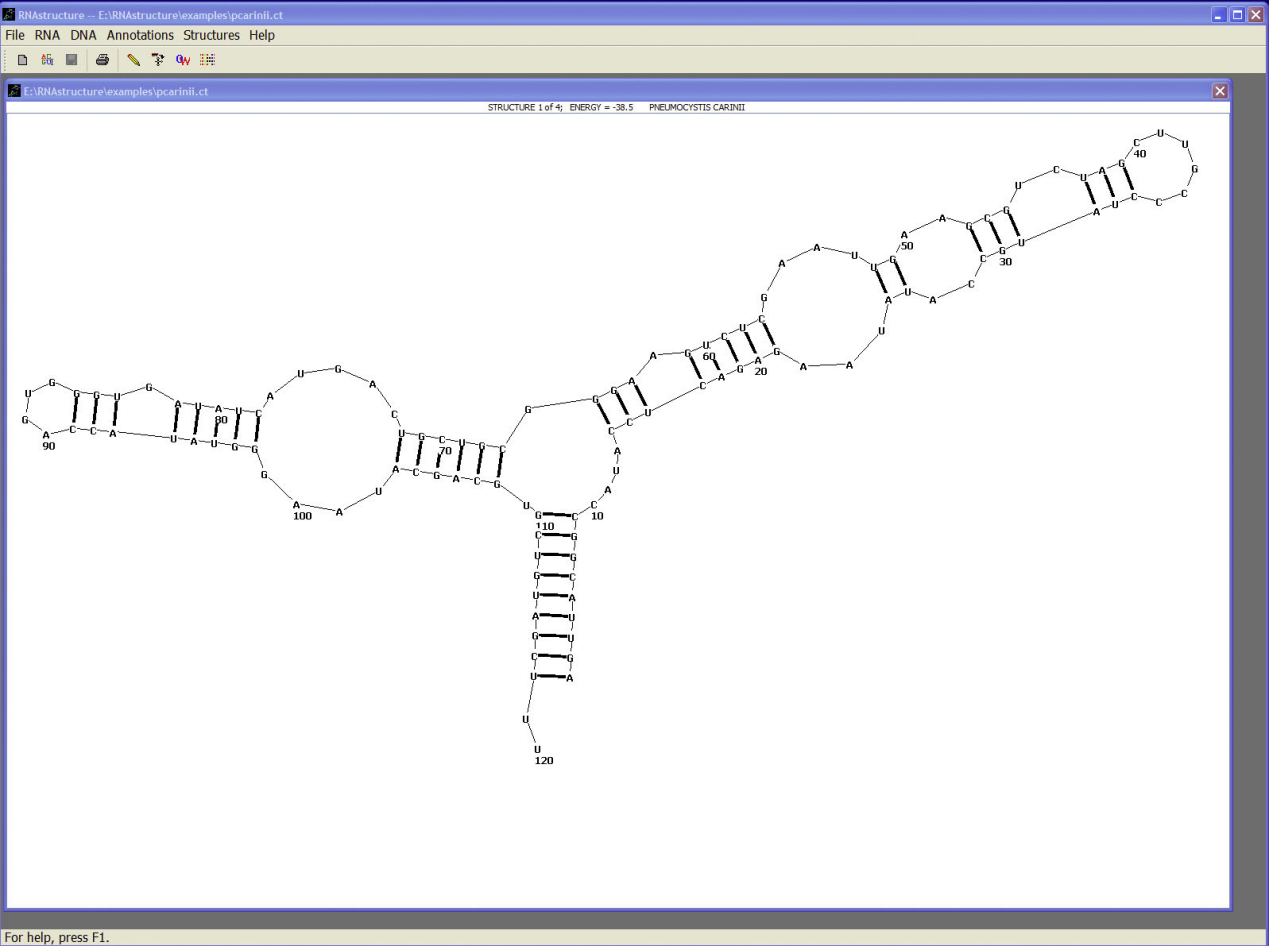
**A screenshot of the JAVA GUI displaying the predicted lowest free energy structure for the sequence input as shown in Figure 2**.

After clicking "Start," the calculation proceeds and the structure is then drawn as shown in Figure 3. The lowest free energy structure is shown by default and the user can display other structures by selecting the "Draw" menu item or by typing control-up-arrow or control-down-arrow.

The same calculation could have been performed on the command line using the program "Fold." The command line for the same default method is "Fold pcarinii.seq pcarinii.ct." A set of postscript images of the predicted structure can then be rendered using "draw pcarinii.ct pcarinii.ps."

Behind the scenes, the structure prediction is performed using the RNA class. For this example, the class was instantiated using a constructor that reads sequences: RNA::RNA("pcarinii.seq", 2, true). The integer 2 indicates that the file is a sequence file and the bool true indicates that the sequence is RNA (as opposed to DNA). Structure prediction is then accomplished using RNA::FoldSingleStrand(10, 20, 3), where the parameters that control suboptimal prediction are the Max % Energy Difference, the Max number of structures, and the Window Size, respectively, as appeared in the GUI (Figure 2). The pairing can then be queried using RNA::GetPair or the drawing coordinates can be determined using RNA::DetermineDrawCoordinates.

## Conclusions

RNAstructure is a software package for RNA secondary structure prediction and analysis. It is designed to make algorithms accessible for a variety of user needs. User-friendly GUIs are available for Windows, using native Windows code, and for Linux/Unix and Macintosh OS-X using JAVA. Text interfaces are provided for performing calculations on the command line or for scripting. Finally, a C++ class library is available to implement the algorithms into new programs. The package can be downloaded at http://rna.urmc.rochester.edu/RNAstructure.html.

## Availability and requirements

• **Project name: **RNAstructure, version 5.0 and later

• **Project home page: **http://rna.urmc.rochester.edu/RNAstructure.html

• **Operating system(s):**

Text interfaces: Compilation is platform independent.

Executables are provided for Microsoft Windows and for 32- and 64-bit Linux.

Class library: Compilation is platform independent.

Windows GUI: Windows XP or later.

JAVA GUI: Compilation is platform independent, but requires Sun JDK 1.6 or higher. Executables are provided for Macintosh OS-X (version 10.5 or later) and Linux.

• **Programming language:**

Text interfaces, Class library, Windows GUI: C++ 

JAVA GUI: JAVA

• **Other requirements:**

Windows GUI: Compilation requires Microsoft Foundation Classes (MFC) as found in Microsoft Visual Studio 2005 or later and the Intel C++ compiler.

JAVA GUI: Requires Sun JAVA JDK version 1.6 or later. Recompilation of SWIG interface requires SWIG version 1.3.39 or later.

• **License: **GNU GPL

• **Any restrictions to use by non-academics: **None.

## Abbreviations

GUI: graphical user interface; ncRNA: non-coding RNA; siRNA: Small interfering RNA

## Authors' contributions

JSR wrote the JAVA interface and the new text interfaces. DHM wrote the C++ class library. Both authors contributed to the debugging of the code. DHM drafted the manuscript and both authors contributed to revisions.
